# Supplementary material for: Complexome profiling of the Chlamydomonas psb28 mutant reveals TEF5 as an early PSII assembly factor
Source: Plant Cell. 2025 Mar 17;37(6):koaf055. doi: 10.1093/plcell/koaf055 (PMC12164590; doi:10.1093/plcell/koaf055)
Supplement: koaf055_Supplementary_Data [file koaf055_supplementary_data.zip › psb28_interactive.zip!/plots/intensity/qualityPlots/peptides/_Cre11.g467640.t1.1.html]

### Cre11.g467640.t1.1

Trivial name:   
SeqCoverage [median]: 21.20  
Mass: 13.64 kDa  
  
 

| Sequence | MissedCleavage | Mass [Da] | wtIntensity | muIntensity | Score |
| --- | --- | --- | --- | --- | --- |
| EVEDLVK | 0 | 830.44 | 8427.51 | 6838.17 | 103.29 |
| EVEDLVKYDPAYFPEYAK | 1 | 2175.04 | 1410.45 | 9457.46 | 111.33 |
| TLADYIR | 0 | 850.45 | 5950.52 | 8836.52 | 88.03 |
| YDPAYFPEYAK | 0 | 1362.61 | 4004.75 | 10430.59 | 96.34 |
|  |  |  | 19793.2 | 35562.7 |  |
